# Associations between the consumption of red meat and processed meat and the incidence of colorectal cancer in Asia: a meta-analysis

**DOI:** 10.3389/fmed.2025.1555717

**Published:** 2025-06-09

**Authors:** Zhiyuan Liao, Wenjiang Wu, Shijun Xia, Linchong Yu, Zhigang Xu, Yue Li

**Affiliations:** Shenzhen Hospital (Fu Tian) of Guangzhou University of Chinese Medicine, Shenzhen, China

**Keywords:** colorectal cancer, colon cancer, rectal cancer, red meat, processed meat, meta-analysis

## Abstract

**Objective:**

This study aimed to evaluate the correlation between the consumption of red meat and processed meat and the incidence of colorectal cancer (CRC) in Asia and provide a scientific basis for reducing the incidence of CRC.

**Methods:**

PubMed, Embase, Cochrane Library, and other databases were searched electronically to collect studies on the correlation between the consumption of red meat and processed meat and the incidence of CRC in Asia. After the quality evaluation of the Newcastle–Ottawa scale, meta-analyses of the selected studies were performed using RevMan 5.4.1. The odds ratio (OR) and 95% confidence interval (95% CI) were combined, and the heterogeneity among the included studies was analyzed via sensitivity analysis. *I*^2^ was used to evaluate the heterogeneity among the included studies.

**Results:**

Twelve articles were included, which involved 13,292 and 12,544 cases in the case and control groups, respectively. The results of the meta-analysis revealed that in the study of the correlation between the consumption of red meat and the incidence of colon cancer, the combined OR was 2.14 (*P* < 0.00001); that for the consumption of red meat and the incidence of CRC, the OR was 1.77 (*P* = 0.006); that for the consumption of red meat and the incidence of rectal cancer, the OR was 2.42 (*P* = 0.0009); and that for the consumption of processed meat and the incidence of CRC, the combined OR was 1.51 (*P* > 0.05).

**Conclusion:**

The results suggest that red meat is a risk factor for the incidence of colon, colorectal, and rectal cancers. However, no significant correlation was found between the consumption of processed meat and the incidence of CRC.

## 1 Introduction

Consumption of red meat and processed meat has been identified to increase the risk of certain cancers, particularly colorectal cancer (CRC). CRC is a common malignancy of the digestive tract. It ranks third among global malignancies after lung cancer and female breast cancer and has the second-highest mortality rate worldwide (1). The International Agency for Research on Cancer classifies processed meat as a carcinogen and red meat as a probable carcinogen. The early symptoms of CRC are not typical and do not appear until the middle and late stages, when the treatment and prognosis are poor, seriously affecting the quality of life of the patients and imposing a huge financial burden on the patients and their families.

At present, although many local and foreign studies have examined the correlation between the consumption of red meat and processed meat and the incidence of CRC, the dietary habits of Asian population are significantly different from those of Western population. Asian diets tend to be high in vegetables, soy products, and fish, with relatively low consumption of red meat and processed meat. To further explore the correlation between the consumption of red meat and processed meat and the incidence of CRC in Asia, a meta-analysis of relevant literature was conducted to obtain new insights and provide a scientific basis for reducing the incidence of CRC in Asia.

## 2 Data and methods

### 2.1 Literature search

#### 2.1.1 Search platform

PubMed, Embase, Cochrane Library, CNKI, Wan Fang database, and VIP Chinese database were searched electronically for relevant studies.

#### 2.1.2 Search strategy

Using the combination of subject terms and free terms, the search strategy was determined according to different databases. In Chinese search, “red meat,” “processed meat,” and “colorectal cancer” were the main topics, and “colon cancer,” “colorectal cancer,” and “colorectal cancer” were the free words. In English retrieval, “processed meat,” “red meat,” and “colorectal neoplasms” were selected as the main words. “Neoplasm, Colorectal,” “Colorectal Tumors,” and “Colorectal Cancer” were the free words. After each keyword was retrieved, the search results of all keywords were combined for retrieval. The search period was up to April 11, 2024.

### 2.2 Literature inclusion and exclusion criteria

#### 2.2.1 Inclusion criteria

The inclusion criteria were as follows: (1) all cases referred to patients with CRC diagnosed for the first time in medical institutions; (2) case-control studies; (4) participants were residing in Asia; (4) studies published locally or abroad before April 11, 2024; (5) research results can be extracted or converted into odds ratio (OR), 95% confidence interval (95% CI), and standard error, and (6) studies that examined the correlation between the consumption of red meat and processed meat and the incidence of CRC.

#### 2.2.2 Exclusion criteria

The exclusion criteria were as follows: (1) the research types were review, animal experimental studies, review literature, etc.; (2) the full text is not retrievable; (3) complete or duplicated data cannot be provided; and (4) no control group was employed.

### 2.3 Literature screening, data extraction, and quality evaluation

Studies with obviously inconsistent research contents were excluded by browsing the abstract, studies meeting the inclusion and exclusion criteria were screened by reading the full text, and relevant data were recorded in Excel. Then, two researchers independently evaluated the quality of the studies using the Newcastle–Ottawa scale (NOS), cross-checked it, and negotiated with a third party to resolve any differences. The scale is composed of three parts: selection of exposure and control population, comparability, and evaluation of exposure or outcome, with 8 items. The score ranges from 0 to 9 points.

### 2.4 Statistical analyses

Meta-analysis was performed using Review Manager 5.4.1. The Cochrane Q test was used to analyze the heterogeneity among all studies, and *I*^2^ was used to evaluate the heterogeneity among the included studies. *P* > 0.1 and *I*^2^ < 50% indicated the lack of heterogeneity among the studies, and the fixed-effect model was used to summarize the data. On the contrary, random-effects model was used, and sensitivity analysis or subgroup analysis was performed to analyze the causes of heterogeneity. Funnel plot analysis was used to determine publication bias in the included studies. For studies lacking certain data, data conversion was performed first.

## 3 Results

### 3.1 Literature search results

**FIGURE 1:**
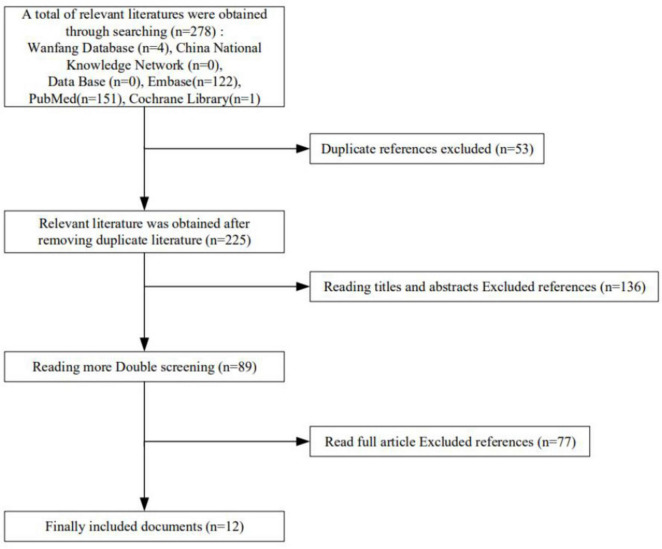
Literature screening flow chart.

A total of 278 studies were retrieved through preliminary database screening. Duplicate studies, reviews, and systematic reviews were excluded. After browsing the titles and abstracts, 189 studies that were obviously different from the subject were excluded, 89 were included in the preliminary screening, 77 were excluded after thorough reading of the full text, and 12 were finally included in the meta-analysis (Figure 1).

### 3.2 Basic characteristics of the included studies

Among the 12 studies included, 11 were case-control studies and 1 was an analytical cross-sectional study. Published from 2010 to 2023, the included studies involved a total of 13,292 cases and 12,544 controls (Table 1).

**TABLE 1:** Basic characteristics of the included studies.

Inclusion studies	Year	District	Research method	Sample size		Sex (male/female)		Outcome index	NOS score
				**Case group**	**control group**	**Case group**	**control group**		
Samarakoon et al. (2)	2018	Sri Lanka	Mismatched case-control study	65	260	28/37	158/102	②	8
Saliba et al. (3)	2019	Israel	Prospective case-control study	5,472	4,554	2,880/2,588	2,389/2,165	② ④	8
Mahfouz et al. (4)	2014	Egypt	Case-control study	150	300	72/78	144/156	② ④	8
Ma et al. (5)	2023	China	Case-control study	2,799	2,799	1,603/1,196	1,603/1,196	① ② ③	9
Alsheridah and Akhtar (6)	2018	Kuwait	Matched case-control studies	103	206	56/47	112/94	②	8
Pramual et al. (7)	2018	Thailand	Analytical cross-sectional study	1,060		339/721		② ④	8
Li et al. (8)	2016	China	1:1 case-control study	400	400	233/167	233/167	② ④	9
Ghrouz and El Sharif (9)	2022	Palestine	Case-control study	105	105	57/48	58/47	② ④	8
Abu Mweis et al. (10)	2015	Jordan	Case-control study	167	240	79/88	108/132	②	9
Song et al. (11)	2019	Korea	Case-control study	703	1,406	480/223	960/446	② ④	9
Promthet et al. (12)	2010	Thailand	Case-control study	130	130	71/59	71/59	①	9
Luo et al. (13)	2019	China	Case-control study	2,138	2,144	1,219/919	1,221/923	① ② ③	9

① Red meat is associated with the incidence of colon cancer. ② Correlation between the consumption of red meat and the incidence of colorectal cancer. ③ Correlation between the consumption of red meat and the incidence of rectal cancer. ④ Correlation between the consumption of processed meat and the incidence of colorectal cancer.

### 3.3 Biased risk assessment of the included studies

The NOS was used to evaluate the quality of the included studies. The NOS scale is suitable for evaluating cohort studies and case-control studies. It has three parts: selection of exposure and control population, comparability, and evaluation of exposure or outcome, with 8 items. The score ranges from 0 to 9 points. The NOS scale was used to evaluate the quality of the 12 studies included, all of which were of high quality (scoring > 7 points).

### 3.4 Results of the meta-analysis

In this study, a meta-analysis was performed on the correlation between the consumption of red meat and processed meat and the incidence of CRC in Asia, and the corresponding forest map was obtained after the heterogeneity test.

#### 3.4.1 Correlation between the consumption of red meat and the incidence of colon cancer

**FIGURE 2:**
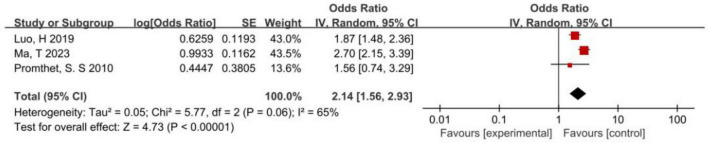
Forest map of correlation analysis between red meat consumption and colon cancer incidence.

The results of the meta-analysis on the correlation between the consumption of red meat and colon cancer incidence were as follows: *P* = 0.06 < 0.1, *I*^2^ = 65% > 50%, suggesting the heterogeneity of the results of the study. Therefore, a random-effect model was selected for the meta-analysis, which revealed that red meat was a risk factor for the incidence of colon cancer (*P* < 0.05). In summary, the risk of colon cancer was 2.14 times that of people who frequently ate red meat in Asia (Figure 2).

#### 3.4.2 Correlation between the consumption of red meat and the incidence of colorectal cancer

**FIGURE 3:**
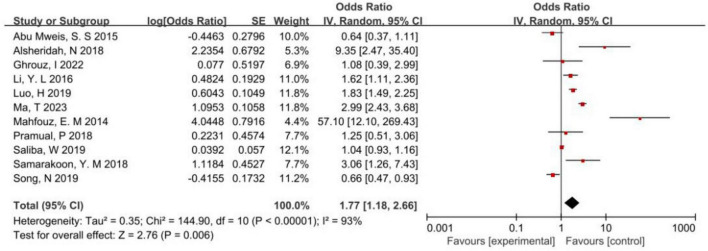
Forest map of correlation analysis between red meat consumption and colorectal cancer incidence.

The results of the meta-analysis on the correlation between the consumption of red meat and the incidence of CRC were as follows: *P* < 0.00001, *I*^2^ = 93% 2 > 50%, suggesting heterogeneity in the results of the study. Therefore, a random-effects model was selected, which revealed that red meat was a risk factor for the incidence of CRC (*P* < 0.05). In summary, the risk of CRC was 1.77 times that of people who frequently ate red meat in Asia (Figure 3).

#### 3.4.3 Correlation between processed meat and incidence of colorectal cancer

**FIGURE 4:**
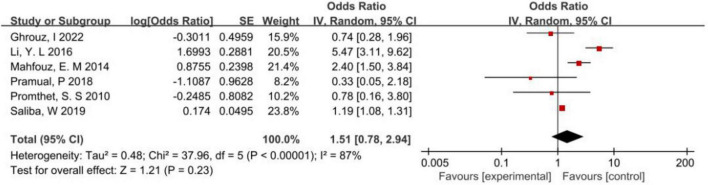
Forest map of correlation analysis between processed meat consumption and colorectal cancer incidence.

The results of the meta-analysis on the correlation between the consumption of processed meat and the incidence of CRC were as follows: *P* < 0.1, *I*^2^ = 87% > 50%, suggesting heterogeneity in the results of the study. Therefore, a random-effects model was selected for meta-analysis, which did not find a significant correlation between the consumption of processed meat and the incidence of CRC in Asia (*P* > 0.05) (Figure 4).

#### 3.4.4 Correlation between the consumption of red meat and the incidence of rectal cancer

**FIGURE 5:**
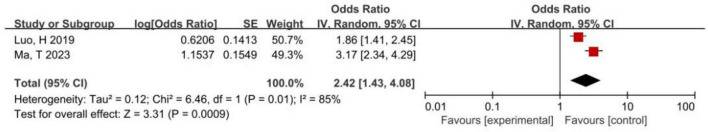
Forest map of correlation analysis between red meat consumption and rectal cancer incidence.

The results of the meta-analysis on the correlation between the consumption of red meat and the incidence of rectal cancer were as follows: *P* = 0.01 < 0.1, *I*^2^ = 85% > 50%, suggesting heterogeneity in the results of the study. Therefore, a random-effects model was selected for meta-analysis, which revealed that red meat was a risk factor for the incidence of rectal cancer (*P* < 0.05). In summary, the risk of CRC was 2.42 times higher in people who frequently ate red meat in Asia than in those who did not eat red meat (Figure 5).

### 3.5 Publication bias

**FIGURE 6:**
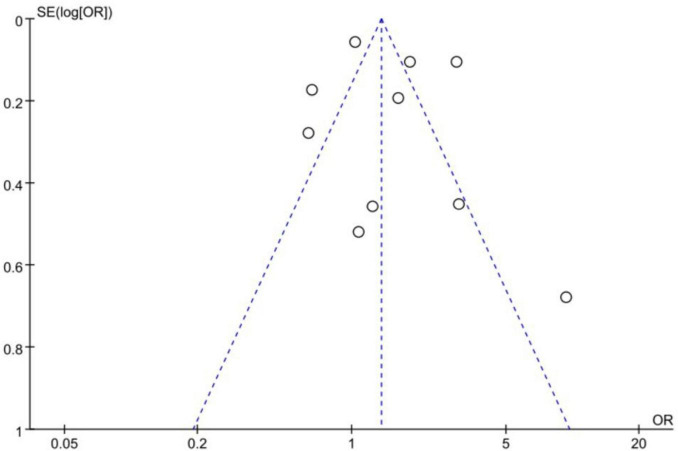
Funnel plot of correlation analysis between red meat consumption and colorectal cancer incidence.

A publication bias analysis was conducted on the studies examining the correlation between the consumption of red meat and the incidence of CRC. The funnel plots were symmetric, suggesting no significant publication bias (Figure 6).

### 3.6 Sensitivity analysis

For the correlation analysis between the consumption of red meat and the incidence of colon cancer, CRC, and rectal cancer, the fixed-effects and random-effects models were used to estimate the combined OR and 95% CI. The combined results of the two models were similar, and the combined results were statistically significant, indicating that the combined results were reliable (Table 2).

**TABLE 2:** Sensitivity analysis of the association between the consumption of red meat and the incidence of colon cancer, colorectal cancer, and rectal cancer in the included studies.

Analysis index	Fixed effect model		Random effects model	
	**OR**	**95% CI**	**OR**	**95% CI**
Association between red meat consumption and colon cancer incidence	2.22	1.89–2.60	2.14	1.56–2.93
Correlation between red meat consumption and colorectal cancer incidence	1.36	1.25–1.47	1.77	1.18–2.26
Association between red meat consumption and rectal cancer incidence	2.37	1.93–2.91	2.42	1.43–0.48

The sensitivity analyses of the correlation between the consumption of red meat and the incidence of CRC and between the consumption of processed meat and the incidence of CRC were performed using the one-by-one elimination method. Studies on the correlation between the consumption of red meat and the incidence of CRC were eliminated one by one, and if the combined results of the remaining studies did not change direction, the study results were robust. Moreover, studies on the correlation between the consumption of processed meat and the incidence of CRC were excluded one after another; consequently, the combined results of the remaining studies were not statistically significant and the direction did not change, indicating that the results were robust, and no significant correlation was noted between the consumption of processed meat and the incidence of CRC (Table 3).

**TABLE 3:** Correlation between red meat consumption and colorectal cancer incidence and between processed meat consumption and colorectal cancer incidence.

	OR (95% CI)	*P*	*I*^2^ (%)
Correlation between red meat consumption and colorectal cancer incidence	1.77 (1.18–2.26)	0.006	93
Excluding Abu Mweis et al. (10)	1.98 (1.29–3.04)	0.002	93
Excluding Alsheridah and Akhtar (6)	1.61 (1.07–2.42)	0.02	93
Excluding Ghrouz and El Sharif (9)	1.84 (1.21–2.82)	0.005	94
Excluding Li et al. (8)	1.81 (1.16–2.84)	0.009	94
Excluding Luo et al. (13)	1.83 (1.13–2.96)	0.01	93
Excluding Ma et al. (5)	1.58 (1.08–2.32)	0.02	89
Excluding Mahfouz et al. (4)	1.50 (1.02–2.21)	0.04	93
Excluding Pramual et al. (7)	1.83 (1.19–2.81)	0.006	94
Excluding Saliba et al. (3)	1.96 (1.22–3.14)	0.006	91
Excluding Samarakoon et al. (2)	1.69 (1.11–2.59)	0.01	94
Excluding et al. (11)	2.01 (1.31–3.08)	0.001	93
Correlation between processed meat consumption and colorectal cancer incidence	1.51 (0.78–2.94)	0.23	87
Excluding Ghrouz and El Sharif (9)	1.71 (0.80–3.65)	0.17	89
Excluding Li et al. (8)	1.22 (0.74–2.01)	0.44	65
Excluding Mahfouz et al. (4)	1.27 (0.52–3.06)	0.60	87
Excluding Pramual et al. (7)	1.73 (0.86–3.47)	0.12	89
Excluding Saliba et al. (3)	1.54 (0.64–3.47)	0.33	80
Excluding Song et al. (11)	1.62 (0.79–3.33)	0.19	89

## 4 Discussion

CRC is a common malignant tumor of glandular epithelial origin, and its incidence increases with age. Many local and foreign studies and reports have examined the risk factors of CRC; however, their results are quite different. Among the 11 articles selected in the meta-analysis of the correlation between the consumption of red meat and the incidence of colon cancer, the combined OR was 2.14 (*P* < 0.00001). In the analysis of the correlation between the consumption of red meat and the incidence of CRC, the OR was 1.77 (*P* = 0.006). In the study of the correlation between the consumption of red meat and the incidence of rectal cancer, the OR was 2.42 (*P* = 0.0009). In the analysis of the correlation between the consumption of processed meat and the incidence of CRC, the combined OR was 1.51 (*P* > 0.05). This suggests that red meat is a risk factor for the incidence of colon, colorectal, and rectal cancers. However, no significant correlation was found between the consumption of processed meat and the incidence of CRC.

This review also revealed that processed meat may be associated with the incidence of colon and rectal cancers in Asia; however, a meta-analysis was not possible because of insufficient literature. Wada et al. (14) showed a positive correlation between the consumption of processed meat and colon cancer risk in men. Oba et al. (15) showed that high consumption of processed meat increased the risk of colon cancer in men.

This meta-analysis had certain limitations. The number of selected articles was small. Only Chinese and English studies with Asian populations as the study participants were selected, and data analysis of large numbers and articles in other languages cannot be performed. For the research results of the meta-analysis of influencing factors, some errors were possible because of the small number of existing studies, which need to be further analyzed. Second, some studies were not included because of the incompleteness of data, which may cause publication bias and affect the combined results. Thirdly, this study mainly relies on case-control studies, lacking cohort studies or RCTs, and fails to account for all potential confounding variables such as genetics, lifestyle, dietary patterns, and other factors, which may reduce the reliability of the research findings. Owing to the limitations of this study, more high-quality controlled studies, cohort studies, and randomized controlled trials (RCTs) are needed to further evaluate the association of red meat and processed meat with the incidence of CRC in Asia.

## Data Availability

The original contributions presented in this study are included in this article/supplementary material, further inquiries can be directed to the corresponding author/s.
